# The Alkalinity of the Blood

**Published:** 1881-08

**Authors:** 


### The Alkalinity of the Blood.

M. J. Garel having made a large number of careful observations with reference to the alkalinity of the blood in different conditions, finds that the results of different observations are not always concurrent, probably due, he says, to imperfections in the process of examination. The following conclusions, however, he feels warranted in advancing with some degree of assurance: The most incontestable fact is the slight alkalinity of the venous blood. I have verified this not only in man, but also in animals. In these last I made parallel analyses of the blood from the femoral artery and vein. The experiment was the more conclusive, as I operated upon a much larger quantity of blood. It is readily perceived, then, how we have noted with M. Lepine that the blood is less alkaline in cyanosed subjects than in the natural state. It is to the increased quantity of carbonic acid that it is necessary to attribute the diminished alkalinity of the blood. In febrile diseases the results obtained are contradictory. There is sometimes increase, sometimes diminution of alkalinity. We are reduced to simple hypotheses to explain these facts. If the alkalinity diminishes, it may be admitted that the globules are more fully charged with carbonic acid. If, on the contrary, there is increase of alkalinity, we suppose that it is in relation to the destruction of discs; the red discs in their destruction yield up potassa to the plasma of the blood. We know, in fact, that the alkalinity of the discs is much greater than that of the serum. This last fact explains how, in the anemic and the cachetic, whose blood is poor in red discs, the alkalinity of the blood is very slight. It accounts, also, for the almost insignificant alkalinity, which I have observed, either in the serum from clots or in different pathological serosities.—*Lyon Med.*—*St. Louis Courier of Medicine.*

